# The Limitations of User-and Human-Centered Design in an eHealth Context and How to Move Beyond Them

**DOI:** 10.2196/37341

**Published:** 2022-10-05

**Authors:** Lex van Velsen, Geke Ludden, Christiane Grünloh

**Affiliations:** 1 eHealth Department Roessingh Research and Development Enschede Netherlands; 2 Department of Communication Science University of Twente Enschede Netherlands; 3 Department of Design, Production and Management University of Twente Enschede Netherlands; 4 Biomedical Systems and Signals group University of Twente Enschede Netherlands

**Keywords:** user-centered design, human-centered design, eHealth, value-sensitive design, citizen science

## Abstract

Human-centered design (HCD) is widely regarded as the best design approach for creating eHealth innovations that align with end users’ needs, wishes, and context and has the potential to impact health care. However, critical reflections on applying HCD within the context of eHealth are lacking. Applying a critical eye to the use of HCD approaches within eHealth, we present and discuss 9 limitations that the current practices of HCD in eHealth innovation often carry. The limitations identified range from limited reach and bias to narrow contextual and temporal focus. Design teams should carefully consider if, how, and when they should involve end users and other stakeholders in the design process and how they can combine their insights with existing knowledge and design skills. Finally, we discuss how a more critical perspective on using HCD in eHealth innovation can move the field forward and offer 3 directions of inspiration to improve our design practices: value-sensitive design, citizen science, and more-than-human design. Although value-sensitive design approaches offer a solution to some of the biased or limited views of traditional HCD approaches, combining a citizen science approach with design inspiration and imagining new futures could widen our view on eHealth innovation. Finally, a more-than-human design approach will allow eHealth solutions to care for both people and the environment. These directions can be seen as starting points that invite and support the field of eHealth innovation to do better and to try and develop more inclusive, fair, and valuable eHealth innovations that will have an impact on health and care.

## Introduction

### Background

For years, user-centered design (UCD) has been considered to be a crucial part of eHealth design. It is believed to improve an innovation’s usefulness and usability [1], to improve end-user satisfaction with the innovation [2], and to increase the quality of user requirements [3]. In addition to this, in the context of eHealth, a strong focus on end users during the design process (patients, care professionals, or others) is deemed to improve adoption rates [4-6], patient decision-making [7], patient engagement [8,9], and patient satisfaction [8]. UCD is a design approach or philosophy that originated in the 1980s. Two seminal publications coined the concept [10] and listed its key principles [11]. According to Gould and Lewis [11], these key principles are that there should be an early focus on users and tasks. First, designers should study the users and the tasks that they need to perform with a technology to understand them fully. Second, the design team should use empirical measurements. Prospective end users need to work with prototypical versions of a technology and their performance and reactions should then be analyzed in a scientific manner. Third, one should apply iterative design. Within the design process, there should be multiple cycles of design, testing, and redesign. Since then, different publications have provided hands-on guidelines on how to implement UCD in practice, for example, an overview by Maguire [12] of the complete UCD process and methods to apply at every stage, and an international standard offering guidance on human-centered design (HCD) activities (International Organization for Standardization 9241-210 “Human-centred design for interactive systems,” the latest version being from 2019 [13]). According to the International Organization for Standardization standard, HCD “is an approach to interactive systems development that aims to make systems usable and useful by focusing on the users, their needs and requirements, and by applying human factors or ergonomics and usability knowledge and techniques.” The international standard outlines the following principles that should be followed in a human-centered approach [13]:

1. The design is based upon explicit understanding of users, tasks, and environments.
2. Users are involved throughout design and development.
3. The design is driven and refined by user-centered evaluation.
4. The process is iterative.
5. The design addresses the whole user experience.
6. The design team includes multidisciplinary skills and perspectives.

Meanwhile, in design research as well as in the field of human-computer interaction (HCI), the term UCD has become a topic of much debate. Although the terms UCD and HCD are often used interchangeably, several authors in these fields have argued that the term UCD reduces a person to someone using a technology, failing to see the whole human being that lives with the technology. Gasson [14], for example, argues for using HCD instead of UCD to avoid a focus on people as technology users and to allow a broader view of human activity supported by technology. In this paper, we will use the term HCD as an umbrella term for both approaches.

HCD has found its way into eHealth design via several road maps that specifically focus on health and well-being as a domain. The Center for eHealth Research (CeHReS) road map [15] is perhaps the most widely used design road map in the field and specifies the following 5 main phases in which HCD is a crucial element: contextual inquiry, value specification, design, operationalization, and summative evaluation. Each phase comes with its own goals and selection of methods that one can apply. The CeHReS road map has been used to guide the development of a wide range of eHealth apps, such as a mobile app to support people in dealing with ticks and tick bites [16], an information dashboard to support nurses in antimicrobial stewardship [17], or a blended exercise therapy intervention for patients with knee and hip osteoarthritis [18]. Other design approaches that heavily hinge on the HCD philosophy and have been used to guide eHealth development include intervention mapping [19], the person-based approach [20], and Integrate, Design, Assess, and Share [21]. Although the authors of these different approaches have all defined their own phases in the design process, their makeup and essence are basically the same. They revolve around extensive end-user (and stakeholder) involvement, iterative design, and working systematically, the same principles that the founders of HCD listed.

Many articles report on the results of HCD processes in eHealth and reflect on the experiences of research and design teams while using the approach (eg, the studies by Fico et al [4], Kramer et al [22], and Atkinson et al [23]). This has led to a body of literature in which the approach receives a lot of praise, with authors claiming that applying the approach has improved the quality of their eHealth service. However, critiquing one’s own approach too much would decrease the value of the design process results as well as the value of the resulting publication. This publication bias may have led the community to believe that HCD is, by definition, the best approach toward eHealth innovation. In addition, this belief in HCD as the best approach has influenced the writers of calls in funding programs (such as the European Union’s Horizon program), in which HCD is often included as a prerequisite for funding. Focusing calls in such a way pushes researchers to use an approach that may not be optimal for their context [24]. In all, it is crucial that the eHealth design community acknowledges the inherent limitations of HCD in eHealth, to (1) reduce the positive bias in their reflections on the application of HCD, (2) raise awareness among design teams about the limitations of this design approach, and (3) improve HCD processes for eHealth by accounting for the limitations of the approach.

### Objectives

In this paper, we aimed to provide an overview of the limitations of using HCD in an eHealth context. These limitations were derived from our own experiences in numerous HCD processes for eHealth services as well as from the larger body of human-centered eHealth design studies. We would like to clarify that we are not opponents of HCD. A simple Google Scholar search of our publication records will show that we have used the approach in the past [17,25,26] and have reflected on its merits. To elaborate on some of our experiences, the second author (GL) and colleagues in the MinD—Designing for people with dementia project reflected on their experiences and on the complexities of involving people with dementia in the design and evaluation of (digital) tools that could improve their psychosocial well-being [27,28]. In another project, we involved children with breathing problems in the design of a smart wearable [29]. Both projects aimed to design for a group of people that was very different from the project team, which called for the inclusion of end-user experiences in the design process. The publications referenced here explain how the teams benefited from working with these groups, creating end results that were (more) acceptable. Nevertheless, in both cases, the teams clearly faced challenges when it came to selection of participants and representation of the complete target group as well as the interpretation of data gathered during cocreation sessions. There were discussions in which the information or knowledge of experts by experience conflicted with related work and knowledge of the project team. In the smart wearable project, we started with including the children who would eventually use the tool in the design process, although child pulmonologists were also involved. A much larger and more diverse group of stakeholders, including child physiotherapists, is involved in a currently running follow-up to this project. This greatly adds to the complexity of decision-making, as was also confirmed in another study in which the third author (CG) was involved. This study concluded that when multiple groups of stakeholders are involved, more knowledge is needed on how to deal with conflicting perspectives [30].

Although we are convinced that HCD approaches are necessary in the design of eHealth innovations, we think that it would be healthy for the community if critical reflection on using HCD becomes common practice, with the ultimate goal of improving our use of HCD. Of course, we are not the first (nor will we be the last) to critically reflect on the concept of HCD. Therefore, before listing the limitations that we would like to stress, we summarize some of the critical reflections on HCD that have been published in the past.

### Previous Reflections on HCD

In his 2005 essay, “Human-Centered Design Considered Harmful,” Norman [31] reflects on some of the principles that underlie HCD; for example, the principle that technology should always adapt to people and not the other way around. He posits that this principle is not really true as people indeed adapt to technology; moreover, technology changes and continues to change our behaviors and our lives. In fact, in eHealth innovation, providing a health intervention that changes people’s behavior or their perspective on health and even their lives is often the aim. According to Norman [31], improving some aspects for individuals or groups may worsen them for others, and the focus on humans and their needs distracts from other design-related activities and may lead to incoherent and complex designs. Norman [31], therefore, suggested an activity-centered design approach, which includes a deep understanding of people but also fosters a deep understanding of technology, tools, and the reasons for the activities.

In 2011, a study by Bannon [32] revisited the roots of the HCI discipline to argue that HCI should develop an even more human-centered approach. For instance, a focus on augmenting people’s existing skills goes beyond merely *considering the user* (and their requirements) and instead, also prioritizes the understanding of people, their concerns, activities, and, in particular, their values and more fundamental needs when designing new technology [32]. Forlizzi [33] urges moving beyond UCD toward stakeholder-centered and service design. Similar to Bannon [32], Forlizzi [33] also reflects on how drastically the field of HCI and also technology and society have evolved, broadening the focus of HCI from ergonomics and usability to also include experience, engagement, and entertainment. Forlizzi [33] identified the lack of an economic perspective in UCD approaches; this perspective is needed, given that today technologies are increasingly being designed as services used by multiple stakeholders. Hence, she urges the HCI community to move beyond UCD and to consider a service design approach that also includes economics [33]. More recently, the top-down approach of HCD as being traditionally led by professionals has been criticized, inviting the exploration and discussion of *community-driven design* [34,35]. The argument goes that today’s global challenges deal with complex sociotechnical systems that require a bottom-up approach in which communities themselves take the lead to solve problems collaboratively, facilitated by professionals [34,36]. The value of actively involving citizens and communities as coresearchers is also well known in approaches such as participatory action research and citizen science [37-39].

These reflections on HCD show that the primary focus on “knowing the end user” is too narrow, and extensions or new approaches have been developed to respond to the need for including additional perspectives and dimensions. Although it incorporates some of the views mentioned previously, this paper specifically focuses on health and well-being as an application domain and the limitations of HCD that we experienced and observed in previous eHealth projects.

## Limitations of HCD in eHealth

### Limitation 1: HCD Tends to Lead to Sampling Bias

“It’s not for me, but my neighbor would love this” is what we often hear participants say during design sessions. It makes one wonder, if we always hear this, are we designing something that nobody wants? Or are we talking to the wrong people? HCD methods most often rely on studying a relatively small sample in depth; this approach is prone to a range of sampling biases. By default, we are not talking to the right people, as not everybody can join the sessions; some specific groups will not be represented at all, whereas other groups are overrepresented (a selection bias) [40]. For example, in a case study by Haslwanter et al [41] that aimed to design a product that enables older adults to stay independently at home for longer, certain methods such as inviting the target audience to a demonstration house led to a recruitment bias in terms of gender, level of mobility, and interest in technology. This sampling bias is further compounded by the difficulty of finding people who are willing and able to spend their time in an interview or design activity. Participating in a design study can be quite time-consuming, meaning that patients need to combine this with their (high) disease burden. Jongsma and Friesen [42] pointed out that participatory research is either too demanding and therefore unfeasible or too uninclusive and therefore unfair. In other words, you sometimes have to take what you get at the cost of biasing your sample (self-selection bias). This bias has been made explicit for experimental research in HCI, which tends to be biased toward younger, more tech-savvy, and more educated participants [43]. For design activities, it is not clear what characterizes those that are more willing to join design activities. In addition, it is also possible, especially among professional participants, that one person participates on behalf of a department or professional group. This person may or may not be chosen by management and acts as a barrier toward involving other people within that group (gatekeeper bias). One could argue that these biases are not a large issue, as qualitative research does not strive toward generalizability, but if we want to include a diverse range of views and contexts, we should strive toward some form of generalizability [44], and we must therefore take these biases seriously. Finally, when biased end-user input is the only source of inspiration for a new eHealth intervention, the implication is that the biased design input is translated into a biased technology. Many authors acknowledge this bias in the Limitations sections of their articles but do not discuss how this bias affected the design or in what way they tried to negate this bias in the design or evaluation phase. Could it be that the design research community has just accepted this bias and its consequences as facts of life?

We would advise design researchers to go further than just naming sampling bias as a limitation in their publication and being done with it. In addition, mapping exactly what the bias looks like (ie, who the most important excluded groups are) and stating how the neglected groups will be included in the design process (eg, in an additional round of design activities using a method that is especially suited for these groups) or in the evaluation activities (eg, targeting the sample of a prototype evaluation toward these groups) would be a great improvement.

### Limitation 2: End-User Input Might Be Biased and Limited

The premise of HCD is that listening to end users and incorporating their needs and wishes into eHealth design will ensure an innovation that end users will want and can use. However, listening to end users (patients or care professionals) has its limitations. First, a lot of the knowledge, opinions, or attitudes that are crucial for eHealth service design are tacit. Tacit knowledge is developed from direct experience and action and is highly pragmatic, and subconsciously understood [45]. Tacit knowledge has been found to be of paramount importance for care professionals for developing working routines [46,47]. Similarly, patient end users (in either preventive or curative care) will have internalized routines and assumptions that they rely on. The challenge with tacit knowledge is that it is difficult to verbalize, and thus elicit, during HCD [48]. Most HCD studies tend to overly rely on traditional interviews and focus groups. These methods are well suited to pose direct questions but are therefore limited in their capacity to elicit tacit knowledge (unless combined with other methods, such as observations). To solve this issue, more creative methods should be applied that have the power to elicit tacit knowledge indirectly, or that allow for the researcher to determine tacit knowledge or procedures for themselves. Two such methods are narrative inquiry [49] and the critical decision method [50]. Next, patients and care professionals apply work-arounds to get things or to get their work done [51,52]. Although this breaking out of protocol might be considered undesirable, it might also be necessary to achieve the best possible outcome for a patient. For example, Yang et al [53] describe the case of a hospital information system that recommends medication dosages, in which physicians override the system as it does not properly take into account pediatric dosages. Furthermore, Dannecker et al [54] describe a myriad of work-arounds that patients with osteoarthritis apply in order to construct pain intensity ratings. On the one hand, work-arounds are a fantastic source of inspiration for design. On the other hand, they can also be a problem as patients or care professionals may not want to disclose them because they are breaking the rules or protocols while implementing them. Zheng et al [55] provide an overview of these challenges and ways to overcome them.

### Limitation 3: HCD Tends to Lead to Overreliance on (Fresh) End-User Input

#### Overview

Consulting the end user early on and throughout the development process of an eHealth service is an important principle of HCD. However, end-user input (or stakeholder input, for that matter; Limitation 4: End users Are Only a Subset of the People Who Should Be Heard During eHealth Design section) is not necessarily the only source of input for designers. The actual design of a service is a creative process that can be fueled by *end-user input* (which can be translated into requirements), but it can also be served by the knowledge and skills of a (multidisciplinary) design team and its creativity, or a by *technology push*.

#### End-User Input

In many articles that describe the HCD process of an eHealth service, end-user input seems to outweigh all the other elements that should inform a thorough design process. We stated in the Introduction section that HCD is part of many (if not most) current eHealth innovation projects. This has created a body of knowledge on user needs and requirements, but this body of knowledge is rarely used to inform other projects. Instead, every project runs its own interview, focus group, and design sessions and the secondary use of end-user input is disregarded. There are some recent exceptions in the field of designing for dementia that resulted in design tools that can, for some part, take over the contextual inquiry phase (such as the MinD toolkit [56]). These largely evolved because of the difficulties of working with this end-user group, but similar tools might be helpful for other patient groups and could well reduce the burden on patients. In addition to this, many designers of eHealth tools are involved in a series of eHealth innovations, in many cases making them experienced “understanders” of particular patient groups. Creativity, so it is argued, is a valuable means of design for solving ill-defined problems. It can drive both the instrumental and hedonic aspects of an innovation in terms of functionality, safety, usability, and affect [57]. A thorough understanding of the implementation context and of end users’ needs and wishes is paramount for creative design [58], and thus, HCD can play a very valuable role in preparing the stage for creative thinking.

*Technology push* seems to have become a dirty word in the eHealth community, and user input, translated into requirements, has become the driving force in many eHealth development processes. This preference for end-user input over propagating technological innovations touches upon the classic debate of the technology push versus the demand pull. Can end users imagine what they actually want, or can they only repeat what they have already seen? Even if people are encouraged to imagine functions that they would like to see in a product or service, this may then relate to *imagined* needs instead of actual user needs. This imposes the risk that these functions are not used in the future [41]. Furthermore, what is the most successful innovation strategy, developing what the market wants, or creating what is technologically possible? Although there are different camps in the scientific community with regard to this issue, it seems like technology push and demand pull are dependent on each other for developing a valuable and successful innovation. Although a technology push is often considered to be the core source of innovation, a demand pull can also drive innovation by bringing forth new ideas and concepts from the users and their context and is always necessary for ensuring economic viability [59]. It seems that, rather than figuring out which approach is best, we should investigate how both approaches can be combined [60].

In line with current open science approaches, design (research) teams should make more efforts to make end-user input reusable and to reuse it where possible. Moreover, rather than thinking that end-user input is the only source of inspiration that can lead to value-adding eHealth innovations, they should aim to find the sweet spot where end user consultation, technology push, and the design team’s knowledge and creativity coincide or come together right. Or, as Norman [31] put it, “Paradoxically, the best way to satisfy users is sometimes to ignore them.”

### Limitation 4: End Users Are Only a Subset of the People Who Should Be Heard During eHealth Design

End users of eHealth solutions are most often patients, care professionals, and citizens, and are crucial in terms of being taken into account when designing a new eHealth service. However, there are also other organizations and actors involved, that is, all stakeholders. Stakeholders can be classified as being either direct or indirect [61]. Direct stakeholders are individuals or organizations who interact directly with the system, whereas indirect stakeholders are affected by the use of the system. Although indirect stakeholders do not interact with the technology themselves, they can exert influence over an innovation or experience consequences from its implementation and use. For example, patients as indirect stakeholders are often not considered when developing electronic medical records, even though the records are about them [62]. Similarly, in the case of developing an eHealth service that gives patients access to their records in Sweden, the “medical profession was not really perceived as a legitimate actor in the development process” [63]. At the same time, the medical profession (clinicians and nurses) in this particular case contested the very idea of the project and was not interested in participating in the design process [64], which created another challenge.

It is imperative to consult both end users and other stakeholders in order to develop a service model and a business model. A service model is an overview of how a technological service interacts with end users and stakeholders, as well as with any other services (on the web or offline). As such, it is a combination of the patient journey and the care protocol (or care path) envisioned for an eHealth service [64]. A business model, on the other hand, is an overview of how the eHealth service is being brought to the market and how it is envisioned to sustain. Both the service and the business model are of paramount importance for creating an eHealth service that is durable and that will be accepted beyond its end users.

Holistic design, in which end users (primary and secondary), lead users, and other parties that can exert influence over the implementation and success of an eHealth service are involved, and in which technology, a service model, and a business model are developed simultaneously, is increasingly being used as a successor to HCD. It is at the core of the CeHReS road map, and in recent years, many developers and researchers have reported their experiences with holistic design (and the CeHReS road map) in case studies (eg, the study by van Velsen et al [25]). Holistic design, in its turn, has disadvantages and challenges that design teams will have to deal with (such as ensuring the collaboration of health professionals [65] and ensuring proper expectation management among all stakeholders [66]). The involvement of different stakeholder groups can provide challenges in terms of balancing their influence as well as the potential assumptions that user groups have of each other that might be based on stereotypes [41,67].

### Limitation 5: Understanding the Added Value of HCD Is Complicated

In general, HCD is considered to be a valuable approach that results in better eHealth services, the point of reference here being eHealth services that are developed without user involvement in any form. This is supported by literature claiming, for example, that user involvement was positive overall and through intermediate factors such as better user requirements [68] or by the revised version of a website based on user input being preferred [7]. A systematic mapping study showed that user participation and involvement can have a positive effect on system success (eg, user satisfaction, ease of use, and system use), but it has also been shown to have negative correlations with system success in older studies [2]. It has been acknowledged that measuring user participation is complex; there is no common conceptual model to measure and validate this effect [2] and we do not have a complete understanding of how user involvement affects product development [68]. In practice, the conclusion that HCD leads to better eHealth services is made through a subjective reflection on the design process by the authors of an article describing the design process. Owing to the competitive nature of academia and the need to publish (or perish), researchers are subject to a (subliminal) bias and are prone to being overly positive about their results [69]. This may mean that our general opinion about HCD is based on a large body of subjective viewpoints.

So, how can we make an objective assessment of the value of HCD for the design of eHealth innovations? If we would fall back on the traditional means to assess the quality of an intervention in health care, then the logical thing to do would be to create a single design briefing, give it to one design team that will apply HCD and another design team that will apply an alternative design process that does not include end users directly and evaluate the resulting eHealth services in terms of usefulness, innovativeness, and usability. This way, we would be able to compare whether one approach “performs” better than the other. However, such studies are difficult to perform (as one has to duplicate the design process) and comparing one “condition” with the other is difficult, as there is also creativity and skill involved in design. Controlling for creativity and skill within the comparison among design approaches would be challenging and maybe even impossible. Despite all the challenges involved in setting up a fair comparison between HCD and an alternative approach, Guo et al [70] conducted a survey study among 389 Chinese digital start-ups and found that applying either a customer orientation or a technology orientation could lead to successful business models. However, combining the 2 approaches led to troublesome situations, as resources were limited, and it was difficult to combine the business logics involved.

The easiest solution toward “unbiasing” our understanding of the value of HCD in the eHealth context would be to adopt a critical view toward the value of the approach in different case studies even if this comes at the cost of a critical peer review. For example, Kip et al [71] critically reflected on their design activities for developing a virtual reality application for practicing coping skills for clients in forensic mental health care and provided an overview of the suitability of different HCD methods for the target population (including both successes and failures). Only by publishing our failures and critical reflections can we create a proper and more nuanced view on the value of HCD (methods) and help eHealth innovation to mature as a research field.

### Limitation 6: HCD Risks of Supporting the Status Quo

When it comes to developing innovative and disruptive eHealth services, questions posed to prospective end users are naturally going to be hypothetical. So, responses are likely be limited to end users’ ability to envision new concepts (More Than Needs and Wishes section). Although it is the role of the designer to develop new concepts, when we try to understand people’s needs, this is more easily done in the current context and not in the future context for which the design is to be developed. Take the example of developing a technology for the prediction of exacerbations based on real-world data and using these predictions for shared decision-making between patients and professionals. This future scenario is so far removed from the current care setting that it is difficult for people to reflect and articulate associated needs. Sometimes, the future scenario is not very far in the future, but the imagined needs do not necessarily reflect the actual needs when it comes to that specific situation. This was illustrated in the study by Haslwanter et al [41] in which there was a difference in what people wanted while seeing the demonstration house and what people then used when implemented in practice. The well-known colloquialism, “what people say they do vs what they actually do” comes to mind, which conveys what we argue to be an even greater challenge when it comes to future scenarios.

Although this limitation might be overcome by not relying solely on end-user input, there is also the risk that incorporating end-user input might hamper innovation. Indeed, it might lead to concluding that the status quo is the most desirable future. For example, research related to patients reading their electronic health records showed that health care professionals question the abilities of patients to understand these records and voice concerns which do not necessarily materialize [30,72]. Here, the desired status quo was a cumbersome process with patients asking for permission to access their paper-based records. It might seem trivial that one stakeholder group is not able to assess the needs of another group; however, health care professionals as domain experts are often considered to be an authority when designing eHealth systems [30]. Similarly, ageism (whereby people hold prejudices about older adults) is often used to think about older adults’ use and ability to use new technology. How aging is framed (eg, as a “problem” to be managed by technology [73]) can also represent common stereotypes and limit design opportunities. As a result, design teams are reluctant to introduce new (technological) concepts, as the general opinion is that older adults do not want change and are unable to deal with new innovations. However, when Jung and Ludden [74,75] interviewed older adults with mobility impairments and presented them with the prospect of using exoskeleton technology, a technology that they were completely unfamiliar with, they seemed rather open to this possible future. But generally, it seems easier for people (patients or health care professionals) to imagine barriers than to imagine opportunities for developing a new type of care, working routine, or society. Consequently, in HCD, we have the tendency to design something new for the current world, rather than designing a new world.

In order to move beyond our prejudices and current (working) routines, there are several things we can do, but these require us to change *how* we do HCD. In their discussion of designing against the status quo, Khovanskaya et al [76] offered several pieces of advice. Designers will need to study and understand the history behind the current situation and the prejudices therein. Then, in order to envision a new reality, designers might need to resort to different sources of inspiration, besides end-user input, such as feminist and queer theory, art, or the maker culture. The trick for the design team will then be to introduce these (disruptive) new ideas to potential end users and stakeholders and to create a safe space in which these ideas can be presented and discussed. Designing against the status quo might mean designing for the long term. The health care setting is conservative and reluctant to change. Therefore, combining short-term ambitions and design ideas (closer to the status quo) with long-term ambitions and design ideas (closer to the disruptive vision) is an approach that is most likely to succeed.

### Limitation 7: Traditional HCD and Designing for Behavior Change Are Not a Good Match

With the increasing importance of preventing chronic diseases and improving lifestyle in general, many eHealth services aim to change the behavior of end users. They must support people to quit smoking, to sit less, or to eat healthier. This trend has led to a research discipline called persuasive design or design for behavior change. Persuasive design is concerned with developing technology “to reinforce, change or shape attitudes or behaviors, or both, without using coercion or deception” [77] and has been found to increase compliance with eHealth services [78]. In design for behavior change, a range of tools and methods have been developed with specific attention to the eHealth context [79]. Although from a normative standpoint, persuading people to perform certain health-improving behaviors might be desirable, it does infringe on the person’s autonomy. For example, a study in the context of smoking cessation showed that although a person might want to stop smoking, they still might not want to make a commitment to behavior change [80]. In a discussion on the ethics of persuasive design; therefore, Berdichevsky and Neuenschwander [81] posit one golden rule for persuasive design, which is as follows: “The creators of a persuasive technology should never seek to persuade a person or persons of something they themselves would not consent to be persuaded to do.” However, the rise of monitoring and coaching technology and the need to make the population adopt a healthier lifestyle have created a situation in which many technologies are being developed that aim to persuade people to adopt a certain behavior *eventually*, while also applying an HCD approach. In this case, however, it is impossible to question potential end users (through interviews, focus groups, and design sessions) about this future goal. Their *initial* standpoint toward a change in behavior may be negative, although at the same time they may have a positive attitude toward caring about (and monitoring) their health. For example, one can probe how one should persuade or support patients with diabetes to be more physically active, but if the participant is unmotivated to do so (eg, the participant is perfectly happy with their current lifestyle), every question or probe is likely to result in a negative reply, if not an aversion to the design session in itself, or could lead to a socially accepted reply (not reflecting the participant’s attitude) just to be over and done with the session. The problem here is that the (technological) solution direction of the design team conflicts with the person’s wishes, desires, or values.

In short, persuasive design and HCD seem to form an unhappy marriage. Therefore, if one were to design a technology that aims to induce health behavior change, one might best trade 1 of the 2 in for something else. Instead of persuasive design, one could resort to using *tuning* as a paradigm that focuses on building internal self-knowledge and self-awareness by supporting appropriate knowledge, skills, and practice [82]. Instead of a single-factor health guidance (eg, to walk 10,000 steps a day), this approach acknowledges the complexities of health in terms of an individual’s context and other behaviors and aims to “support a person gaining knowledge, skills, and practice of how to tune their health across contexts” [82]. In addition, taking into account the end user’s stage of chance (following the transtheoretical model [83]) in eHealth design and personalization will ensure that content, functionalities, and design strategies [84] are geared toward the aspects of behavior change to which the end user is most receptive. If one is quite attached to persuasive design, one could trade HCD in for value-sensitive design (VSD). Rather than focusing on what persuasive technology should do and how (as one would do in HCD), VSD aims to understand why a design might be harmful, and it will reveal the value conflicts or tensions that must be solved [85]. The latter approach will respect the participants and their context and will not evoke negative emotions. Once the value conflicts are fully mapped, it will be the design team’s task to create a design that is capable of reaching the behavior change goals while respecting the end users’ values. Or, one could go even one step further and supply VSD with capability sensitive design [86]. In such an approach, the design team has to elicit not only *what* end users value but also whether these outcomes *ought* to be valued.

### Limitation 8: HCD Tends to Miss Out on Ethical, Societal, and Political Aspects

HCD activities focus on individual users, their context, and their needs and expectations in relation to specific tasks and goals. Thus, HCD tends to prioritize the microlevel rather than the mesolevel and macrolevel. However, organizational aspects on the mesolevel are crucial when it comes to the implementation of eHealth solutions in real life (End users Are Only a Subset of the People Who Should Be Heard During eHealth Design section). HCD supports the economic and social pillars of sustainability [13]. However, ethical, societal, and political issues on the macrolevel can be overlooked when focusing on the individual user.

Technological advancements such as machine learning and artificial intelligence (AI), have the potential to support people in their everyday tasks (eg, decision support tools in health care). However, they also have the potential to increase inequality by amplifying biases and assumptions that are invisible to users. This has been outlined in the book “Weapons of Math Destruction,” where mathematical models and algorithms are typed as *opaque* (lacking transparency or completely invisible), *damaging* (harmful or unfair for certain people and creating pernicious feedback loops), or that *scale* (have the capacity to grow exponentially) [87]. Negative examples include decision support for judges using recidivism models or a university ranking model that creates an ecosystem of education and industry of tutors that adapt to that scoring system [87]. As more and more decisions are automated in the future based on AI, algorithmic biases potentially lead to discrimination based on certain characteristics such as income, education, gender, or ethnicity [88]. Although these decisions may work for many, people who fall outside what has been incorporated in the design as the “norm” (eg, through specific training data or through how the models are designed) have to deal with the consequences without the opportunity to appeal the decisions. Discrimination may stay hidden if we focus on specific user groups and how technology can support their tasks and goals (eg, supporting a judge in sentencing decisions).

These examples show that the human perspective is often not considered or only very narrowly considered for a small user group. The focus on the individual user within an HCD can be complemented by approaches such as VSD, responsible research and innovation [89], or human-centered explainable AI [90], which aim to incorporate ethical and societal aspects into the design process. Within HCD alone, this is difficult to address, especially if the innovations are so complex that it is difficult to communicate the risks or form a black box by definition. Furthermore, as COVID-19 pandemic measures and tracking apps showed, certain decisions include not only a technological, scientific, and societal perspective but also a political one [91,92]. Given the circumstances, political decisions might prioritize certain values over others (eg, public security over privacy in relation to track and trace), this issue is also relevant for eHealth technologies (eg, apps used for contact tracing and risk information [93]).

### Limitation 9: HCD Thinks About the Beginning but Not the End

Most eHealth services that are developed through an HCD approach are accompanied by elaborate onboarding procedures and implementation plans. The desire to reach and secure a high number of end users makes sense, as one of the common key performance indicators for these services is the number of end users served (for a longer period). Interestingly, this focus on the first use of the service seems to come at a cost. It rarely happens that a design process also devotes attention and time to longer-term use or to ending the use of a service. Should an app change at some point in the user’s journey? When has an eHealth service fulfilled its purpose? How do we determine this moment? Which actions are associated with ending the end-user journey supported by the eHealth service? It rarely happens that answers to these questions are sought and processed into service design for the eHealth context.

A topic that is associated with ending the personal use of the eHealth service is ending the eHealth service completely. It may feel a bit contradictory to think about the terminating of a service during the design stage, but for some services, this will be crucial for acceptance and for preventing undesirable situations. The COVID-19 pandemic has made the need for these deimplementation plans very clear. Contact tracing apps were built on top of the privacy-preserving exposure notification frameworks developed by Google and Apple. Despite the fact that these frameworks did not require tracking the geographical location of end users, they were met with a lot of skepticism and privacy concerns. Indeed, one cannot exclude the possibility that tracking the geographical location by means of this technology might be possible in the future. So although the use of these technologies might be legitimate and useful for the short term, they might be harmful in the long run. Therefore, the introduction of eHealth services that come with large implications, such as the COVID-19 contact tracing apps, should be accompanied by a plan that specifies when we can stop using them and how we can erase all the data that they collect during their lifetime.

## Discussion

### Nine Limitations

In this paper, we have described 9 limitations that we currently see with the application of HCD in eHealth. This set of limitations came about by critically reflecting on our own eHealth innovation projects and by reviewing the body of work in this domain. Of course, not all limitations are restricted to the eHealth context and many of them are applicable to the full range of digital services one can develop. However, we felt that it was important to provide a complete overview of the main limitations that we have seen. Again, we would like to emphasize that, even after composing this list of limitations, we do feel that HCD processes have their place in the design of eHealth innovations, especially in combination with other sources of input, such as available knowledge and a technology push. Our objective was to provide a wake-up call to researchers and designers in the eHealth domain. Although some actively seek and implement ways to improve the role of HCD in their innovations, others continue to rely on standard (and suboptimal) ways to involve end users. The most important point we want to make is that critical reflection on applying HCD methods in the design of eHealth services is lacking, and this is not helping the field of eHealth innovation to mature. This list of limitations is most probably not conclusive, and we hope that more critical reflection by other researchers in the field will eventually lead to a better understanding of (1) how and when HCD methods can really contribute to the design process (and when they would not do so); (2) how HCD methods can lead to more generalizable knowledge that the field needs and could share; and (3) how HCD methods can be integrated in the process of multidisciplinary design teams that include relevant health, technological, ethical, and other expertise.

In addition to the limitations, this paper also discusses several current developments and opportunities to improve the use of HCD in the design of eHealth innovations. This means that there are already signs that the field of eHealth innovation is changing and taking important next steps that change how we see and deal with HCD in eHealth innovation. We briefly want to reflect on and elaborate on 3 of these developments here as we see them as very important for the future of eHealth innovation.

### More Than Needs and Wishes

In limitations 4, 7, and 8, we have mentioned how VSD or value-based design and also capability sensitive design can provide guidance in involving multiple stakeholders and integrating ethical perspectives in the design process. VSD defines human values as “what is important to people in their lives, with a focus on ethics and morality” [94]. Although VSD displays some similarities to HCD, it includes aspects that go beyond it as well, such as the commitment to analyze both direct and indirect stakeholders; to distinguish designer values, stakeholder values, and values explicitly supported by the project; to conduct an analysis on individual, group, and societal levels; and the possibility for technology and social structures to coevolve [94]. Hence, VSD may offer a solution to some of the biased or limited views of traditional HCD approaches. For instance, the commitment of a thorough analysis of direct and indirect stakeholders might mitigate the risk of sampling bias (limitation 1) and bias toward and overreliance on end-user input (limitations 2 and 3), as it opens up the design space in terms of who should get a seat at the table. The challenge in applying VSD is that a focus on values can lead to a rather abstract understanding of what end users and other stakeholders consider important. This means that it leaves a serious task for the design team to translate this understanding into a tangible design, a task in which they may well want to involve end users again. We need a good body of work describing and reflecting on the processes used to do this (eg, the studies by Boerema et al [95] and Smits et al [96]).

### More Than Consulting End Users

In limitation 7, we discussed the problem that in some eHealth development processes, the (technological) solution direction of the design team conflicts with a person’s wishes, desires, or values. Limitation 7 goes on to discuss ways to deal with this situation, but it also triggers the question of whether the solution direction that the design team was aiming for was the right one. Were they trying to answer the wrong question all along? An approach that tries to tackle this is citizen science, which seeks to engage “citizens” (ie, everyone who at some point may deal with the outcomes of science) in research in different ways. Citizen science has been around for a while now, and its uptake and importance in health and biomedical research are growing [97]. Citizen science overlaps with HCD in its methods and aims and can range from *contributory* (eg, participation of the public or patients through data collection and processing) to *collaborative* (eg, public involvement in refining research questions, analyzing data, or disseminating findings), *cocreation* (eg, researchers and members of the public working together across key research processes), and *extreme citizen science* (in which researchers provide tools and methods to enable communities to develop their own participatory research projects) [38]. In the health and well-being domain, the people dealing with the outcome of research are often also the subject of the research (patients or experts by experience). A larger adoption of citizen science could, on the one hand, help us in making the transition from seeing patients as subjects whose opinions we politely ask for, to coresearchers who are active in not only providing data or answers to our questions but also in asking the right questions and setting a research agenda for (public) health. On the other hand, citizen science philosophy somewhat conflicts with the issues and recommendations that we mention in limitation 6 (*HCD Risks to Support the Status Quo*). As we discuss in limitation 6, in order to envision a new reality, designers or a design team should bring in inspiration, come up with new ideas and imagine new futures, and find ways to introduce and discuss these with the public or the community they are working with. The 10 principles of citizen science, put forward by the European Citizen Science Association in 2015 [98], provide an initial set of guidelines to take up citizen science in eHealth innovation. It is up to the field of eHealth innovation to further discuss and critically reflect on how and when to use citizen science approaches in the eHealth context.

### More Than Humans

In limitation 8, we discuss how HCD risks missing out on ethical, societal, and political aspects. A prioritization of the microlevel, the personal level, has consequences, especially when this is the preferred and largely dominant approach in a particular field. One such consequence could be that we disregard the impact that our innovations have on the environment. A recent appeal in *The Lancet Digital Health* stressed this very point [99]. In addition, several researchers have called for moving beyond the dominant anthropocentric perspective to include nonhuman perspectives [100,101]. As Giaccardi and Redström [101] describe, we may at some point reach the boundaries of what can be conceived through UCD and HCD processes. The increasing complexity of what we can design and the increasing consequences that our designs can have call for new methods and for reconsideration of the role of HCD methods and the weight given to them.

### Conclusions

In this paper, we have presented 9 limitations of using HCD in eHealth and 3 directions of inspiration to improve design practices. We feel that these directions provide good starting points to do better and to try and develop more inclusive, fair, and valuable eHealth innovations that will have an impact on health and care. We trust and hope that this discussion of limitations as well as this short outlook to the future of eHealth innovation will stir up a bit of dust and would be very happy to see others add to it so that with more careful, considered, and critical use of HCD we can improve our eHealth research and innovation methods together.

## Abbreviations

AI: artificial intelligence
CeHRes: Center for eHealth Research
HCD: human-centered design
HCI: human-computer interaction
UCD: user-centered design
VSD: value-sensitive design

## References

[1] Vredenburg K, Mao J, Smith P, Carey T (2002). A survey of user-centered design practice. Proceedings of the SIGCHI Conference on Human Factors in Computing Systems.

[2] Abelein U, Paech B (2013). Understanding the influence of user participation and involvement on system success – a systematic mapping study. Empir Software Eng.

[3] Kujala1 S (2008). Effective user involvement in product development by improving the analysis of user needs. Behav Inform Technol.

[4] Fico G, Martinez-Millana A, Leuteritz J, Fioravanti A, Beltrán-Jaunsarás ME, Traver V, Arredondo MT (2019). User centered design to improve information exchange in diabetes care through ehealth : results from a small scale exploratory study. J Med Syst.

[5] Marien S, Legrand D, Ramdoyal R, Nsenga J, Ospina G, Ramon V, Spinewine A (2019). A user-centered design and usability testing of a web-based medication reconciliation application integrated in an eHealth network. Int J Med Inform.

[6] Mitzner T, Dijkstra K (2016). Evaluating User Centered Design of E-Health for Older Adults: Using Subjective Methods.

[7] Atkinson NL, Massett HA, Mylks C, McCormack LA, Kish-Doto J, Hesse BW, Wang MQ (2011). Assessing the impact of user-centered research on a clinical trial eHealth tool via counterbalanced research design. J Am Med Inform Assoc.

[8] Nazi KM, Turvey CL, Klein DM, Hogan TP (2018). A decade of veteran voices: examining patient portal enhancements through the lens of user-centered design. J Med Internet Res.

[9] Wachtler C, Coe A, Davidson S, Fletcher S, Mendoza A, Sterling L, Gunn J (2018). Development of a mobile clinical prediction tool to estimate future depression severity and guide treatment in primary care: user-centered design. JMIR Mhealth Uhealth.

[10] Norman D, Draper S (1986). User Centered System Design New Perspectives on Human-Computer Interaction.

[11] Gould JD, Lewis C (1985). Designing for usability: key principles and what designers think. Commun ACM.

[12] Maguire M (2001). Methods to support human-centred design. Int J Human Comput Stud.

[13] (2019). ISO 9241-210:2019 Ergonomics of human-system interaction — Part 210: human-centred design for interactive systems. ISO.

[14] Gasson S (2003). Human-centered vs. user-centered approaches to information system design. J Inform Technol Theory Application.

[15] van Gemert-Pijnen JE, Nijland N, van Limburg M, Ossebaard HC, Kelders SM, Eysenbach G, Seydel ER (2011). A holistic framework to improve the uptake and impact of eHealth technologies. J Med Internet Res.

[16] van Velsen L, Beaujean DJ, Wentzel J, Van Steenbergen JE, van Gemert-Pijnen JE (2015). Developing requirements for a mobile app to support citizens in dealing with ticks and tick bites via end-user profiling. Health Informatics J.

[17] Wentzel J, van Velsen L, van Limburg M, de Jong N, Karreman J, Hendrix R, van Gemert-Pijnen JJ (2014). Participatory eHealth development to support nurses in antimicrobial stewardship. BMC Med Inform Decis Mak.

[18] Bossen D, Kloek C, Snippe HW, Dekker J, de Bakker D, Veenhof C (2016). A blended intervention for patients with knee and hip osteoarthritis in the physical therapy practice: development and a pilot study. JMIR Res Protoc.

[19] Boekhout JM, Peels DA, Berendsen BA, Bolman CA, Lechner L (2017). An eHealth intervention to promote physical activity and social network of single, chronically impaired older adults: adaptation of an existing intervention using intervention mapping. JMIR Res Protoc.

[20] Yardley L, Morrison L, Bradbury K, Muller I (2015). The person-based approach to intervention development: application to digital health-related behavior change interventions. J Med Internet Res.

[21] Mummah SA, Robinson TN, King AC, Gardner CD, Sutton S (2016). IDEAS (integrate, design, assess, and share): a framework and toolkit of strategies for the development of more effective digital interventions to change health behavior. J Med Internet Res.

[22] Kramer L, Blok M, van Velsen L, Mulder B, de Vet E (2021). Supporting eating behaviour of community-dwelling older adults: co-design of an embodied conversational agent. Design Health (Abingdon).

[23] Atkinson NL, Saperstein SL, Desmond SM, Gold RS, Billing AS, Tian J (2009). Rural eHealth nutrition education for limited-income families: an iterative and user-centered design approach. J Med Internet Res.

[24] Hallewell Haslwanter JD, Fitzpatrick G, Miesenberger K (2018). Key factors in the engineering process for systems for aging in place contributing to low usability and success. J Enabling Technol.

[25] van Velsen L, Illario M, Jansen-Kosterink S, Crola C, Di Somma C, Colao A, Vollenbroek-Hutten M (2015). A community-based, technology-supported health service for detecting and preventing frailty among older adults: a participatory design development process. J Aging Res.

[26] Hooglugt F, Ludden GD (2020). A mobile app adopting an identity focus to promote physical activity (MoveDaily): iterative design study. JMIR Mhealth Uhealth.

[27] Lim JN, Almeida R, Holthoff-Detto V, Ludden GD, Smith T, Niedderer K, MinD consortium (2019). What is needed to obtain informed consent and monitor capacity for a successful study involving People with Mild Dementia? Our experience in a multi-centre study. Designing With and for People With Dementia: Wellbeing, Empowerment and Happiness.

[28] Niedderer K, Holthoff-Detto V, van Rompay TJ, Karahanoğlu A, Ludden GD, Almeida R, Durán RL, Aguado YB, Lim JN, Smith T, Harrison D, Craven MP, Gosling J, Orton L, Tournier I (2022). This is Me: evaluation of a boardgame to promote social engagement, wellbeing and agency in people with dementia through mindful life-storytelling. J Aging Stud.

[29] Siering L, Ludden GD, Mader A, van Rees H (2019). A theoretical framework and conceptual design for engaging children in therapy at home-the design of a wearable breathing trainer. J Pers Med.

[30] Cajander A, Grünloh C (2019). Electronic health records are more than a work tool: conflicting needs of direct and indirect stakeholders. Proceedings of the 2019 CHI Conference on Human Factors in Computing Systems.

[31] Norman DA (2005). Human-centered design considered harmful. Interact.

[32] Bannon L (2011). Reimagining HCI. Interact.

[33] Forlizzi J (2018). Moving beyond user-centered design. Interactions.

[34] Hekler E, Taylor J, Dow S, Morris M, Grant F, Phatak S, Norman D, schraefel M, Lewis DM (2019). Proceedings of the Companion Publication of the 2019 on Designing Interactive Systems Conference 2019 Companion.

[35] Norman D, Spencer E (2019). Community-based, human-centered design. jnd.org.

[36] Meyer M, Norman D (2020). Changing design education for the 21st Century. She Ji J Design Econ Innov.

[37] Wilson B (2018). A short history of community-driven design. Resilience for All.

[38] Borda A, Gray K, Downie L (2019). Citizen science models in health research: an Australian commentary. Online J Public Health Inform.

[39] Oberschmidt K, Grünloh C, Nijboer F, van Velsen L (2022). Best practices and lessons learned for action research in eHealth design and implementation: literature review. J Med Internet Res.

[40] Latulipe C, Gatto A, Nguyen H, Miller D, Quandt S, Bertoni A, Smith A, Arcury T (2015). Design considerations for patient portal adoption by low-income, older adults. Proc SIGCHI Conf Hum Factor Comput Syst.

[41] Hallewell Haslwanter JD, Fitzpatrick G (2016). Why do few assistive technology systems make it to market? The case of the HandyHelper project. Univ Access Inf Soc.

[42] Jongsma K, Friesen P (2019). The challenge of demandingness in citizen science and participatory research. Am J Bioeth.

[43] Dickinson A, Arnott J, Prior S (2007). Methods for human – computer interaction research with older people. Behav Inform Technol.

[44] Groger L, Mayberry PS, Straker JK (2016). What we didn’t learn because of who would not talk to us. Qual Health Res.

[45] McAdam R, Mason B, McCrory J (2007). Exploring the dichotomies within the tacit knowledge literature: towards a process of tacit knowing in organizations. J Knowl Manag.

[46] Thornton T (2006). Tacit knowledge as the unifying factor in evidence based medicine and clinical judgement. Philos Ethics Humanit Med.

[47] Kothari AR, Bickford JJ, Edwards N, Dobbins MJ, Meyer M (2011). Uncovering tacit knowledge: a pilot study to broaden the concept of knowledge in knowledge translation. BMC Health Serv Res.

[48] Van Velsen L, Wentzel J, Van Gemert-Pijnen JE (2013). Designing eHealth that matters via a multidisciplinary requirements development approach. JMIR Res Protoc.

[49] Kothari A, Rudman D, Dobbins M, Rouse M, Sibbald S, Edwards N (2012). The use of tacit and explicit knowledge in public health: a qualitative study. Implement Sci.

[50] Okechukwu OJ, Weller G, Watt J (2014). Eliciting experts? knowledge in emergency response organizations. Int J Emergency Services.

[51] Bjørn P, Balka E (2007). Health care categories have politics too: unpacking the managerial agendas of electronic triage systems. Proceedings of the Tenth European Conference on Computer Supported Cooperative Work.

[52] Fitzpatrick G, Ellingsen G (2012). A review of 25 years of CSCW research in healthcare: contributions, challenges and future agendas. Comput Supported Coop Work.

[53] Yang Z, Ng B, Kankanhalli A, Luen Yip JW (2012). Workarounds in the use of IS in healthcare: a case study of an electronic medication administration system. Int J Human Comput Stud.

[54] Dannecker E, Warne-Griggs M, Royse L, Hoffman K (2019). Listening to patients' voices: workarounds patients use to construct pain intensity ratings. Qual Health Res.

[55] Zheng K, Ratwani RM, Adler-Milstein J (2020). Studying workflow and workarounds in electronic health record–supported work to improve health system performance. Ann Intern Med.

[56] MinD tools - Designing for people with dementia homepage. MinD tools - Designing for people with dementia.

[57] Zeng L, Proctor R, Salvendy G (2010). Creativity in ergonomic design: a supplemental value-adding source for product and service development. Hum Factors.

[58] Christiaans H, Venselaar K (2005). Creativity in design engineering and the role of knowledge: modelling the expert. Int J Technol Des Educ.

[59] Di Stefano G, Gambardella A, Verona G (2012). Technology push and demand pull perspectives in innovation studies: current findings and future research directions. Res Policy.

[60] Brem A, Voigt K (2009). Integration of market pull and technology push in the corporate front end and innovation management—insights from the German software industry. Technovation.

[61] Friedman B, Kahn P, Borning A, Huldtgren A (2013). Value sensitive design and information systems. Early engagement and new technologies: Opening up the laboratory.

[62] Friedman B, Kahn JP, Borning A (2006). Value sensitive design and information systems. Human-computer Interaction Management Information Systems: Foundations.

[63] Erlingsdóttir G, Lindholm C (2015). When patient empowerment encounters professional autonomy: the conflict and negotiation process of inscribing an eHealth service. Scandinavian J Public Administration.

[64] Broekhuis M, Weering MD, Schuit C, Schürz S, van Velsen L (2021). Designing a stakeholder-inclusive service model for an eHealth service to support older adults in an active and social life. BMC Health Serv Res.

[65] Noergaard B, Sandvei M, Rottmann N, Johannessen H, Wiil U, Schmidt T, Pedersen SS (2017). Development of a web-based health care intervention for patients with heart disease: lessons learned from a participatory design study. JMIR Res Protoc.

[66] van Dooren MM, Siriaraya P, Visch V, Spijkerman R, Bijkerk L (2019). Reflections on the design, implementation, and adoption of a gamified eHealth application in youth mental healthcare. Entertainment Comput.

[67] Grünloh C, Myreteg G, Cajander Å, Rexhepi H (2018). "Why do they need to check me?" patient participation through eHealth and the doctor-patient relationship: qualitative study. J Med Internet Res.

[68] Kujala S (2003). User involvement: a review of the benefits and challenges. Behav Inform Technol.

[69] Fanelli D (2010). Do pressures to publish increase scientists' bias? An empirical support from US States data. PLoS One.

[70] Guo H, Wang C, Su Z, Wang D (2020). Technology push or market pull? Strategic orientation in business model design and digital start‐up performance*. J Prod Innov Manag.

[71] Kip H, Kelders SM, Bouman YH, van Gemert-Pijnen LJ (2019). The importance of systematically reporting and reflecting on eHealth development: participatory development process of a virtual reality application for forensic mental health care. J Med Internet Res.

[72] Delbanco T, Walker J, Bell SK, Darer JD, Elmore JG, Farag N, Feldman HJ, Mejilla R, Ngo L, Ralston JD, Ross SE, Trivedi N, Vodicka E, Leveille SG (2012). Inviting patients to read their doctors' notes: a quasi-experimental study and a look ahead. Ann Intern Med.

[73] Vines J, Pritchard G, Wright P, Olivier P, Brittain K (2015). An age-old problem. ACM Trans Comput Hum Interact.

[74] Jung M, Ludden G (2018). Potential of exoskeleton technology to assist older adults with daily living. Proceedings of the Extended Abstracts of the 2018 CHI Conference on Human Factors in Computing Systems.

[75] Jung MM, Ludden GD (2019). What do older adults and clinicians think about traditional mobility aids and exoskeleton technology?. J Hum Robot Interact.

[76] Khovanskaya V, Dombrowski L, Harmon E, Korn M, Light A, Stewart M, Voida A (2018). Designing against the status quo. interactions.

[77] Oinas-Kukkonen H, Harjumaa M (2008). Towards deeper understanding of persuasion in software and information systems. Proceedings of the First International Conference on Advances in Computer-Human Interaction.

[78] Kelders SM, Kok RN, Ossebaard HC, Van Gemert-Pijnen JE (2012). Persuasive system design does matter: a systematic review of adherence to web-based interventions. J Med Internet Res.

[79] Niedderer K, Clune S, Ludden G (2017). Design for Behaviour Change Theories and Practices of Designing for Change.

[80] Ploderer B, Smith W, Howard S, Pearce J, Borland R (2012). Things you don't want to know about yourself: ambivalence about tracking and sharing personal information for behaviour change. Proceedings of the 24th Australian Computer-Human Interaction Conference.

[81] Berdichevsky D, Neuenschwander E (1999). Toward an ethics of persuasive technology. Commun ACM.

[82] schraefel M, Hekler E (2020). Tuning. interactions.

[83] Prochaska JO, Velicer WF (1997). The transtheoretical model of health behavior change. Am J Health Promot.

[84] Ludden G, Hekkert P (2014). Design for healthy behavior: design interventions and stages of change. Proceedings of the 9th International Conference on Design and Emotion.

[85] Davis J (2009). Design methods for ethical persuasive computing. Proceedings of the 4th International Conference on Persuasive Technology.

[86] Jacobs N (2020). Capability sensitive design for health and wellbeing technologies. Sci Eng Ethics.

[87] O'Neil C (2016). Weapons of Math Destruction How Big Data Increases Inequality and Threatens Democracy.

[88] Ferrer X, Nuenen TV, Such JM, Cote M, Criado N (2021). Bias and discrimination in AI: a cross-disciplinary perspective. IEEE Technol Soc Mag.

[89] Owen R, Macnaghten P, Stilgoe J (2012). Responsible research and innovation: from science in society to science for society, with society. Sci Public Policy.

[90] Ehsan U, Riedl M (2020). Human-centered explainable AI: towards a reflective sociotechnical approach. Proceedings of the HCI International 2020 - Late Breaking Papers: Multimodality and Intelligence: 22nd HCI International Conference, HCII 2020.

[91] Greenhalgh T, Ozbilgin M, Contandriopoulos D (2021). Orthodoxy, illusio, and playing the scientific game: a Bourdieusian analysis of infection control science in the COVID-19 pandemic. Wellcome Open Res.

[92] Meyer J, Kay J, Epstein D, Eslambolchilar P, Tang L (2020). A life of data: characteristics and challenges of very long term self-tracking for health and wellness. ACM Trans Comput Healthcare.

[93] Dubov A, Shoptawb S (2020). The value and ethics of using technology to contain the COVID-19 epidemic. Am J Bioeth.

[94] Friedman B, Hendry D (2019). Value Sensitive Design Shaping Technology with Moral Imagination.

[95] Boerema S, van Velsen L, Vollenbroek-Hutten M, Hermens H (2017). Value-based design for the elderly: an application in the field of mobility aids. Assist Technol.

[96] Smits M, Ludden G, Peters R, Bredie S, van GH (2022). Values that matter: a new method to design and assess moral mediation of technology. MIT Press Direct.

[97] Wiggins A, Wilbanks J (2019). The rise of citizen science in health and biomedical research. Am J Bioeth.

[98] Gold M (2019). ECSA 10 Principles of citizen science. OSF Home.

[99] Chevance G, Hekler EB, Efoui-Hess M, Godino J, Golaszewski N, Gualtieri L, Krause A, Marrauld L, Nebeker C, Perski O, Simons D, Taylor JC, Bernard P (2020). Digital health at the age of the Anthropocene. Lancet Digital Health.

[100] Coulton P, Lindley J (2019). More-than human centred design: considering other things. Design J.

[101] Giaccardi E, Redström J (2020). Technology and more-than-human design. Design Issues.

